# Comorbidities in the UK Primary Sjögren’s Syndrome Registry

**DOI:** 10.3389/fimmu.2022.864448

**Published:** 2022-04-22

**Authors:** Jessica Tarn, Dennis Lendrem, Michael Barnes, John Casement, Wan-Fai Ng

**Affiliations:** ^1^Translational and Clinical Research Institute, Newcastle University, Newcastle upon Tyne, United Kingdom; ^2^Centre for Translational Bioinformatics, Queen Mary, University of London (QMUL), London, United Kingdom; ^3^National Institute for Health and Care Research (NIHR) Newcastle Biomedical Research Centre & National Institute for Health and Care Research (NIHR) Newcastle Clinical Research Facility, Newcastle upon Tyne Hospitals NHS Foundation Trust, Newcastle upon Tyne, United Kingdom

**Keywords:** Sjogren’s syndrome, comorbidity, crossectional analysis, polypharmacy, stratified medicine

## Abstract

**Introduction:**

Primary Sjögren’s Syndrome (PSS) is a chronic disease characterised by symptoms of oral and ocular dryness, pain, fatigue, anxiety and depression. PSS patients can be subclassified by the pattern of severity of these five key symptoms using the Newcastle Sjögren’s Stratification Tool (NSST). Although PSS is often associated with one or more comorbidities, the relationship between comorbidities, polypharmacy, and PSS symptom burden is unclear. Using data from the UK Primary Sjögren’s Syndrome Registry (UKPSSR) we describe the landscape of polypharmacy and comorbidities in PSS.

**Methods:**

The UKPSSR is research biobank of clinically well-defined PSS patients where clinical, demographic, comorbidities and concomitant medications data are recorded. Patients were subclassified into the four NSST subgroups: Low Symptom Burden (LSB), High Symptom Burden (HSB), Dryness Dominated Fatigue (DDF) and Pain Dominated Fatigue (PDF). Group analyses of comorbid conditions and polypharmacy scores were performed. Comorbidity and Polypharmacy Scores (CPS) were modelled as a function of age, sex, symptom duration, body mass index (BMI), current immunosuppressant and hydroxychloroquine prescriptions and NSST subgroup.

**Results:**

There were marked differences in the number and the nature of comorbidities associated with the NSST subgroups. LSB and DDF patients were characterized by fewer comorbidities and medications. In contrast, HSB and PDF patients were associated with more comorbidities and were more likely to be prescribed multiple medications. Group analysis shows that HSB patients are more closely associated with peripheral vascular disease and infection whereas the PDF patients were associated with cardiovascular disease and gastrointestinal comorbidities. Comorbidity and polypharmacy scores increase with age and BMI regardless of symptom subgroup and symptom duration. In addition, the longer the reported symptom duration the higher the associated comorbidities and polypharmacy scores.

**Conclusion:**

Comorbid conditions are more prevalent in some subgroups of the PSS cohort but increase with age and BMI across the entire cohort. It is unclear from these data whether specific comorbid conditions are a consequence of PSS or represent shared aetiology or pathogenetic susceptibility. Regardless, these findings may have implications for disease management and clinical trial design.

## Introduction

Primary Sjögren’s syndrome (PSS) is a complex immune-mediated inflammatory disease (IMID) which is associated with a significant negative impact on health-related quality of life (1). PSS presents with a female:male ratio of 9:1 and has a bimodal age distribution with peaks at ages 20-30 and 50-70 (2). PSS is a systemic disease where extra-glandular involvement is common (3). Multimorbidity is also common in PSS (4–6) however studies of comorbid conditions in pSS are relatively scarce. Our understanding of comorbidities in PSS is confounded by the heterogeneity of the disease in terms of symptom burden and disease activity, moreover, variables such as age, BMI and socioeconomic factors need to be considered also. PSS poses considerable health costs which are compounded by multimorbidity which is a barrier to effective disease management strategies and therapeutic development. For this reason, it is essential we have a more complete view of the comorbid conditions associated with PSS.

Although some comorbid conditions have been studied in the context of PSS in isolation such as cardiovascular disease and gastrointestinal disease (7–10), few studies have examined the range of comorbid conditions within PSS cohorts. A 2010 study explored the profile of comorbidities in a Taiwanese PSS cohort in comparison to a healthy cohort (6). Several conditions were significantly associated with PSS in this study; including cardiac arrythmias, hyperlipidaemia, migraine, fibromyalgia, asthma, pulmonary circulation disorders, hypothyroidism, liver disease, peptic ulcers, deficiency anaemias, depression and psychoses. Similarly, another study of comorbidities in Sjögren’s syndrome (SS) compared to matched controls reported increased prevalence of hypertension, osteoarthritis, osteoporosis and depression (5). More recently Pego-Regiosa and colleagues reviewed the most common comorbid conditions associated with PSS including cardiovascular disease, musculoskeletal conditions, infections and non-haematological malignancies (4). There are clues therefore to the most prevalent conditions associated with PSS however it is unclear whether there are subgroups of PSS patients and comorbid conditions that may occur together or share risk factors.

It is well known that the burden of PSS symptoms varies between individuals, with some PSS sufferers struggling significantly with their symptoms and others managing their condition well (11). This is reflected in measures of health-related quality of life such as EQ-5D utility (1).

Recently we identified distinct subgroups within the UKPSSR cohort, demonstrated biological differences between those subgroups and developed a stratification tool (NSST) (11). NSST uses patient reported symptom scores for dryness, fatigue, pain, anxiety and depression to assign pSS sufferers into four symptom burden-based subgroups, namely the High Symptom Burden (HSB), Low Symptom Burden (LSB), Dryness Dominant Fatigue (DDF) and Pain Dominant Fatigue (PDF) subgroups. Patients in the HSB subgroup report high scores for all 5 symptoms, LSB group patients report low scores for all symptoms, PDF group patients report high scores for dryness, pain and fatigue and DDF group patients report high scores for dryness and fatigue. We demonstrated that the four groups have different profiles of gene expression and serum proteins therefore it is feasible that these groups represent true subgroups of the disease. Personalised medicine strategies in PSS aim to improve disease management and clinical trial design by targeting therapeutics to individuals who are most likely to benefit or have the most improvements to gain.

Here, we explore the comorbidities associated with PSS using data from the United Kingdom Primary Sjögren’s Syndrome Registry (UKPSSR). The UKPSSR is a well characterised cohort of individuals with PSS from 32 centres across the UK and contains detailed prospective data regarding comorbid conditions and medications for over 900 participants. We investigate the profile of comorbid conditions within the UKPSSR and assess differences between the symptom-based subgroups we previously identified.

## Methods

Data were collected from the UK Primary Sjögren’s Registry (UKPSSR, http://www.sjogrensregistry.org) between August 2009 and December 2018 (n = 931) (12). This time interval limits the cohort analysed within this study to data from the registry that was fully curated at the time of analysis, which includes having complete data for the 5 NSST symptom domains necessary for symptom burden group classification. All participants fulfil the American European Consensus Group classification criteria (13). Data were collected prospectively using standardised proforma at the time of clinic appointment. For all patients attending the regional PSS clinic, the following data are collected as part of their care: EULAR Sjögren’s Syndrome Disease Activity Index (ESSDAI) (14), EULAR Sjögren’s Syndrome Patient Reported Index (ESSPRI) (15), Hospital Anxiety and Depression Scale (HADS) (16), EQ-5D UK utility (http://www.euroqol.org), Profile of Fatigue (Pro-F) (17). Baseline polypharmacy and comorbidity data were available from the UKPSSR all participants. The following variables were available at baseline for all subjects: Age, Disease Duration, Sex, Ro/La status, ESSDAI, ESSPRI, HADS, EQ-5D UK utility, Pro-F.

Polypharmacy Score was calculated as the sum of medications at the time of clinic appointment. Details of comorbid conditions were collected prospectively, standardised using ICD10 codes, and converted to clinically meaningful categories using Clinical Classifications Software Refined (CCSR) tools. A Chronic Conditions Indicator score (CCI) was calculated using CCSR tools which was used for downstream analyses. Comorbidity-Polypharmacy Score (CPS) was calculated as the sum of polypharmacy score and CCI score. Group analyses of significant differences were performed using analysis of variance (ANOVA) and pairwise comparisons were performed using Tukey-Kramer analysis. CPS score was modelled using symptom burden subgroup, current hydroxychloroquine use, current immunosuppressant use (Cyclosporine, Azathioprine, Leflunomide, Methotrexate, Mycophenylate, Infliximab, Rituximab, Etanercept), age, disease duration and BMI to identify significant factors associated with CPS score. Statistical analysis was performed in R and JMP (18, 19).

## Results

**Figure 1:**
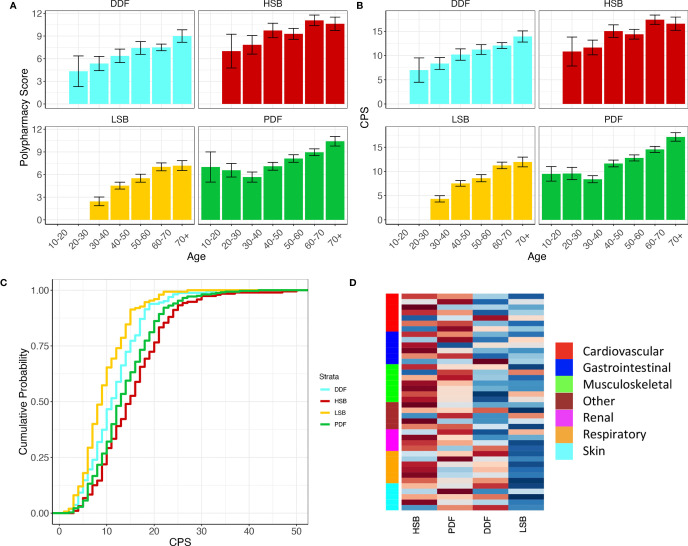
Polypharmacy score **(A)** and CPS score **(B)** increase with age in all symptom burden subgroups. Cumulative distribution plot showing higher CPS in the HSB and PDF groups compared to the LSB and DDF groups **(C)**. **(D)** Heatplot of the proportions of the top 40 comorbidities (ICD10) in the four subgroups. The heatplot cells are scaled row-wise where colour scale is representative of the proportions of each comorbidity separately across the four subgroups. A darker red colour represents a higher proportion and darker blue colour represents a lower proportion. Side bar colours denote a representative category for each comorbidity. For full annotation see **Supplementary Figure S1**.

Summary data for the entire cohort and for the four symptom subgroups are presented in **Tables 1.1**, **1.2** respectively. The data show that the number of chronic conditions and number of medications increase significantly with age in all symptom subgroups (**Figures 1A, B**, both p<0.0001) which was expected. CPS is significantly different between the four subgroups (**Figure 1C**, ANOVA: p<0.0001). Pairwise comparisons show that CPS is lowest in the LSB subgroup then the DDF and PDF subgroups and highest in the HSB subgroup (**Figure 1C**, p<0.05 for all comparisons).

**Table 1.1:** Cohort characteristics: a summary of key demographic and clinical data for 931 PSS participants at time of recruitment.

**Sex**	864f/67m	
**Symptom Burden Group**	DDF 180, HSB 218, LSB 165, PDF 368	
	***Mean (sd)***	***Median (25th, 75th centile)***
*Age*	58.1 (12.8)	60.0 (50.0, 67.0)
*BMI*	26.6 (5.6)	25.4 (22.6, 29.3)
*ESSDAI Score*	4.8 (5.0)	3.0 (1.0, 7.0)
*ESSPRI Score*	5.4 (2.2)	5.7 (4.0, 7.0)
*Disease Duration (years)*	6.1 (5.8)	4.1 (1.7, 8.7)
*Polypharmacy Score*	8.1 (4.8)	7.0 (4,.0 11.0)
*CCI*	4.7 (2.8)	4.0 (3.0, 6.0)
*CPS*	12.8 (6.6)	12.0 (8.0, 17.0)

**Table 1.2:** Cohort characteristics: a summary of key demographic and clinical data for four symptom burden groups of PSS participants at the time of recruitment.

*Sex*	*DDF*	*172f/8m*	
	HSB	202f/16m	
	LSB	148f/17m	
	PDF	342f/26m
	***Symptom Burden Group***	***Mean (sd)***	***Median (25th, 75th centile)***
*Age*	DDF	58.8 (13.0)	62.0 (49.8, 68.0)
	HSB	57.0 (11.9)	58.0 (49.0, 65.0)
	LSB	57.9 (12.2)	60.0 (49.0, 67.0)
	PDF	58.6 (13.4)	61.0 (51.0, 68.0)
*BMI*	DDF	25.7 (5.3)	24.5 (22.2, 28.3)
	HSB	27.3 (6.4)	26.6 (22.8, 30.7)
	LSB	26.0 (5.6)	24.9 (21.8, 28.0)
	PDF	26.9 (5.3)	26.1 (22.9, 29.6)
*Disease Duration*	DDF	6.6 (6.4)	4.1 (2.0, 9.7)
	HSB	5.9 (5.8)	4.2 (1.5, 7.9)
	LSB	6.1 (5.5)	4.1 (1.6, 9.6)
	PDF	6.0 (5.6)	4.1 (1.7, 8.3)
*ESSPRI*	DDF	5.3(1.3)	5.0 (4.3, 6.0)
	HSB	7.0 (1.7)	7.0 (6.0, 8.3)
	LSB	2.0 (0.9)	2.0 (1.3, 2.7)
	PDF	6.1 (1.5)	6.0 (5.0, 7.3)
*ESSDAI*	DDF	4.4 (4.7)	3.0 (1.0, 7.0)
	HSB	5.4 (5.0)	4.0 (2.0, 8.0)
	LSB	3.5 (4.6)	2.0 (0.0, 5.0)
	PDF	5.1 (5.1)	4.0 (2.0, 7.5)
*Polypharmacy Score*	DDF	7.3 (4.1)	7.0 (4.0, 10.0)
	HSB	9.6 (5.5)	9.0 (5.0, 13.0)
	LSB	5.9 (3.7)	5.0 (3.0, 8.0)
	PDF	8.4 (4.7)	8.0 (5.0, 11.0)
CCI	DDF	4.1 (2.5)	4.0 (2.0, 6.0)
	HSB	5.3 (3.1)	5.0 (3.0, 7.0)
	LSB	3.5 (2.1)	3.0 (2.0, 5.0)
	PDF	5.1 (2.8)	5.0 (3.0, 7.0)
*Hydroxychloroquine (current)*	DDF	55/180 (31%)	
	HSB	94/218 (43%)	
	LSB	50/165 (30%)	
	PDF	154/368 (42%)	
*Immunosuppressants (current)*	DDF	21/180 (12%)	
	HSB	40/218 (18%)	
	LSB	15/165 (9%)	
	PDF	55/368 (15%)	

The most common comorbidities associated with PSS in this cohort irrespective of symptom burden group are osteoarthritis (36%), gastro-oesophageal reflux disease (31%), essential hypertension (20%), chronic cystitis (10%), hypercholesterolaemia (10%), asthma (9%), osteoporosis (8%), fibromylalgia (8%), irritable bowel syndrome (8%) and chronic ischaemic heart disease (5%).

When analysing the top 50 comorbidities in this cohort (**Figure 1C** and **Supplementary Figure S1**) we found that the prevalence differs depending on subgroup. Broadly, the LSB and DDF subgroups have fewer associated comorbidities overall whereas the HSB and PDF subgroups have more (p <0.0001). The HSB and PDF subgroups also have differences in the prevalence of certain comorbid conditions (**Figure 1D**). Within the musculoskeletal domain the HSB subgroup proportionally has higher numbers of comorbidities such as spondylopathies, musculoskeletal pain and fibromyalgia, whereas osteoporosis and osteoarthritis are found at proportionally higher levels in the PDF group. In addition, chronic obstructive pulmonary disease, abdominal hernia, and hypertension are more prevalent in the PDF subgroup than the HSB subgroup, whereas peripheral vascular disease, urinary tract infections, depressive disorders, nerve root disorders and gastroduodenal ulcers are more prevalent in the HSB subgroup. Of the comorbidities associated with the LSB subgroup, musculoskeletal pain and osteoporosis are found at the highest proportions. Within the DDF subgroup respiratory disease and cardiac dysrhythmias are found at the highest proportions. LSB patients rarely report comorbidities associated with skin or respiratory disease.

We modelled CPS score using other variables from the cross-sectional dataset to determine whether there are any clinical variables that have a strong relationship with CPS score. Symptom burden group, current hydroxychloroquine use, current immunosuppressant use, higher age, higher BMI and longer disease duration are significantly associated with higher CPS score (p < 0.05 for all).

## Discussion

The influence of comorbid conditions in PSS is still unclear and poses challenges for clinical management of the disease as well as therapeutic development and personalised medicine strategies. Health related quality of life has been shown to be lower in individuals with PSS and is a key metric for quality adjusted life year (QALY) measurements used in cost-benefit analyses. In addition, it is unclear whether some symptoms associated with PSS are purely a symptom of the disease or a consequence of comorbid conditions or polypharmacy.

We report marked differences in comorbidities and polypharmacy across the PSS subgroups. In line with previous work, we also show that PSS patients have increased comorbid conditions and medications with age: intercepting this adverse trajectory is a key challenge. Taking the cohort as a whole the current findings replicate the data published by other groups. The most common comorbidities in this cohort are within the musculoskeletal category such as osteoarthritis, osteoporosis and fibromyalgia. Another common category is cardiovascular disease or risk factors associated with it such as chronic ischaemic heart disease, hyperlipidaemia and hypertension. Gastrointestinal disease such as reflux and IBS is another common comorbidity within this cohort.

The symptom-based subgroups appear to capture some of the variability in individual differences in comorbid conditions and polypharmacy that present with PSS. Firstly, there is a clear and significant difference between the subgroups in terms of CPS score, with the LSB group having the lowest CPS score and HSB the highest (**Figure 1C**). CPS scores increase with age in all subgroups, but differences between subgroups are consistent across age groups.

HSB and PDF patients associate with a wide range of comorbidities across all disease categories however there are differences between these groups. Hypertension and COPD appear more prevalent in the PDF group whereas peripheral vascular disease and infections are more prevalent in the HSB group, amongst others. Several studies have identified that PSS patients have a higher risk for cardiovascular disease (8–10, 20), it is possible that these patients are more likely to be in the HSB group. The comorbidities associated with the LSB and DDF groups appear to be more limited. This heterogeneity between the subgroups suggests that the development of multiple comorbid diseases is linked to factors associated with PSS and its patient subgroups. This observation could have important implications for stratified medicine and clinical trial design, where the heterogeneity of PSS patient cohorts presents a major barrier to understanding response to treatments. It is unclear however whether PSS is the instigating factor in the development of these comorbidities either due shared pathophysiology or factors associated with susceptibility such as genetic or environmental influences. Alternatively, the treatments used, and lifestyle adaptations required by the patients to manage the symptoms of PSS could impact the development of comorbid conditions. For example, it has been suggested that the use hydroxychloroquine may have a protective effect against cardiovascular disease in PSS (21). As these data are cross-sectional it is difficult to determine any cause and effect.

This study has limitations; it is based on cross-sectional data from a national cohort and the findings require validation in international cohorts. Despite the relatively large sample size of the UKPSSR cohort, many comorbid conditions are present at such a low frequency that a much larger cohort or meta-analysis is required to analyse the full spectrum of diseases associated with PSS. In addition, long-term follow up would be essential to enable causal analysis of comorbid conditions, in which the sequence of comorbid diagnoses could provide interesting insights to pathophysiology of the disease.

## Data Availability Statement

The raw data supporting the conclusions of this article will be made available by the authors, without undue reservation.

## Ethics Statement

Research ethics approval was granted by the UK North-West Research Ethics Committee. The patients/participants provided their written informed consent to participate in this study.

## Author Contributions

JT - formal analysis, original draft writing, manuscript review and editing. DL – conceptualisation, formal analysis, original draft writing, manuscript review and editing. MB – data interpretation. JC- data interpretation. W-FN – conceptualisation, funding acquisition, data interpretation manuscript review and editing. All authors contributed to the article and approved the submitted version.

## Funding

This work was supported by Foundation of Research in Rheumatology (FOREUM): (grant number: 022). This work received infrastructure support from the National Institute for Health Research (NIHR) Newcastle Biomedical Research Centre and the NIHR Clinical Research Facility, based at Newcastle upon Tyne Hospitals NHS Foundation Trust and Newcastle University; and from the Arthritis Research UK and Versus Arthritis Newcastle upon Tyne Experimental Arthritis Treatment Centre. The UKPSSR received grant support from the UK Medical Research Council (grant number G0800629 to W-FN, SJB, BG) and British Sjögren’s Syndrome Association. This work was supported, in part, by the National Institute for Health Research: NIHR202635 - Artificial Intelligence Multimorbidity Development Award and the Innovative Medicines Initiative 2 Joint Undertaking (JU) (NECESSITY grant agreement No 806975) receiving support from the European Union’s Horizon 2020 research and innovation program and EFPIA.

## Conflict of Interest

W-FN has undertaken clinical trials and provided consultancy or expert advice in the area of Sjögren’s syndrome to the following companies: GlaxoSmithKline, MedImmune, UCB, Abbvie, Roche, Eli Lilly, Takeda, Resolves Therapeutics, Sanofi, Novartis and Nascient.

The remaining authors declare that the research was conducted in the absence of any commercial or financial relationships that could be construed as a potential conflict of interest.

## Publisher’s Note

All claims expressed in this article are solely those of the authors and do not necessarily represent those of their affiliated organizations, or those of the publisher, the editors and the reviewers. Any product that may be evaluated in this article, or claim that may be made by its manufacturer, is not guaranteed or endorsed by the publisher.
