# Supplementary material for: Comorbidities in the UK Primary Sjögren’s Syndrome Registry
Source: Front Immunol. 2022 Apr 22;13:864448. doi: 10.3389/fimmu.2022.864448 (PMC9116135; doi:10.3389/fimmu.2022.864448)
Supplement: Supplementary file 1 [file DataSheet_1.docx]

Supplementary Materials

*Supplementary Figure S1. Heatplot of the proportions of the top 40 comorbidities (ICD10) in the four subgroups with annotated ICD10 codes. The heatplot cells are scaled row-wise where colour scale is representative of the proportions of each comorbidity separately across the four subgroups. A darker red colour represents a higher proportion and darker blue colour represents a lower proportion. Side bar colours denote a representative category for each comorbidity.*
